# Clinical Insights into the Neurodevelopmental Impact of 16p CNVs in an Italian Clinical Cohort

**DOI:** 10.3390/genes17020247

**Published:** 2026-02-21

**Authors:** Ilaria La Monica, Maria Rosaria Di Iorio, Antonia Sica, Lucio Pastore, Barbara Lombardo

**Affiliations:** 1 Department of Molecular Medicine and Medical Biotechnologies, Federico II University, Via Sergio Pansini 5, 80131 Naples, Italy; lamonica@ceinge.unina.it (I.L.M.); sicaa@ceinge.unina.it (A.S.); lucio.pastore@unina.it (L.P.); 2 CEINGE-Biotecnologie Avanzate Franco Salvatore, Via G. Salvatore 486, 80145 Naples, Italy; diiorio@ceinge.unina.it

**Keywords:** neurodevelopmental disorders, copy number variants, chromosome 16p11.2, 16p13.3, 16p13.11, a-CGH, genotype–phenotype correlation, Italian cohort

## Abstract

**Background**: Neurodevelopmental disorders (NDDs) are a heterogeneous group of conditions characterized by cognitive, behavioral, and developmental impairments, frequently linked to structural genomic alterations. Copy number variants (CNVs) involving chromosome 16, particularly the short arm 16p, are recognized contributors to neurodevelopmental variability. Despite increasing international evidence, data from Italian clinical cohorts are still limited. **Methods**: We investigated 1200 patients referred for genetic evaluation due to suspected NDDs, including autism spectrum disorder (ASD), intellectual disability (ID), global developmental delay, and language impairment. All individuals underwent array comparative genomic hybridization (a-CGH) analysis, and identified variants were correlated with detailed clinical, cognitive, and behavioral assessments. The analysis focused on recurrent CNVs at 16p11.2, 16p13.3, and 16p13.11, regions containing dosage-sensitive genes relevant to neurodevelopment. **Results**: CNVs involving the 16p region were identified in 96 patients (8% of the cohort), encompassing both deletions and duplications. Deletions were mainly associated with developmental delay, language deficits, and ASD-related features, whereas duplications were more frequently linked to behavioral dysregulation, attentional deficits, and variable cognitive impairment. Marked phenotypic variability was observed among individuals carrying similar CNVs, suggesting the contribution of modifying genetic or environmental factors. In a subset of patients, additional CNVs were identified, potentially exacerbating clinical severity, consistent with the two-hit model. **Conclusions**: This study confirms a strong association between recurrent 16p CNVs and a wide spectrum of neurodevelopmental phenotypes in an Italian clinical cohort. The findings emphasize the diagnostic utility of systematic genomic screening and the importance of an integrated genotype–phenotype approach to improve clinical interpretation, management, and genetic counseling in NDDs.

## 1. Introduction

Neurodevelopmental disorders (NDDs) constitute a heterogeneous spectrum of neurological conditions characterized by cognitive impairments, behavioral abnormalities, and deficits in adaptive functioning, affecting approximately 1–3% of the population and showing a higher prevalence in males [1]. Advances in genomic technologies have established a major genetic contribution to NDDs, with both rare and common variants influencing neurodevelopmental traits [2,3,4,5]. Among these, copy number variants (CNVs) represent a substantial source of genetic risk, particularly in clinically complex and sporadic cases. CNVs, defined as deletions or duplications of specific genomic regions, frequently encompass dosage-sensitive genes involved in neurodevelopmental and synaptic pathways. Recurrent CNVs arising from nonallelic homologous recombination are of clinical relevance, as they are observed in a wide phenotypic outcome, including intellectual disability (ID), psychomotor developmental delay (PDD), autism spectrum disorder (ASD), language delay, and broader NDD phenotypes [6,7,8,9,10,11,12,13,14,15,16,17,18,19]. A major challenge in clinical genetics is the interpretation of CNVs in the context of incomplete penetrance, variable expressivity, and inheritance status [4,20,21]. Identical CNVs may be associated with markedly different phenotypic outcomes, ranging from severe neurodevelopmental impairment to apparently unaffected carrier parents, complicating genotype–phenotype correlations and genetic counseling [19,22,23,24]. This phenotypic variability emphasizes the need for studies aimed at refining the clinical significance of recurrent CNVs.

Among these, chromosome 16, particularly its short arm (16p), represents a highly informative model for studying the contribution of structural genomic variation to NDDs [23]. Approximately 10% of the 16p sequence (~7.8 megabase pairs, Mb) consists of segmental duplications, predisposing this region to recurrent rearrangements mediated by nonallelic homologous recombination (NAHR) [25,26,27,28]. Consequently, CNVs involving recurrent loci within 16p, including 16p11.2, 16p13.1, and 16p13.3, are among the most frequently reported structural variants in individuals with ASD, ID, PDD, language delay, and other NDDs, with prevalence estimates ranging from 0.3% to nearly 1% in affected cohorts [23,29,30,31,32,33,34,35,36,37]. At these loci, recurrent breakpoints (BPs) defined by flanking segmental duplications give rise to CNVs with relatively consistent genomic boundaries, allowing correlations between BP architecture and neurodevelopmental phenotypes to be explored [25,26,34]. For example, CNVs involving the canonical BPs at 16p11.2 have been repeatedly associated with ASD, ID, language impairment, and variable neuropsychiatric manifestations, supporting a dosage-sensitive model of disease risk linked to this region [12,19,27,30,31,36]. Similar breakpoint-mediated mechanisms have been described for CNVs at 16p13.1 and 16p13.3, further emphasizing the importance of breakpoint structure in modulating phenotypic expressivity [38,39].

Phenotypically, duplications are more often associated with attention deficit-hyperactivity disorder (ADHD), tremors, and behavioral dysregulation, with an increased risk for psychosis and severe ID. Conversely, deletion carriers are linked to ASD, speech delay, and language impairment [40,41,42,43,44,45]. This region therefore offers a valuable framework for investigating how recurrent CNVs contribute to neurodevelopmental phenotypic diversity through mechanisms of dosage sensitivity, penetrance, expressivity, and breakpoint architecture [46,47]. Despite extensive international data, CNVs affecting chromosome 16p remain insufficiently characterized in Italian clinical cohorts, where population-specific genetic backgrounds, referral patterns, and diagnostic practices may influence variant interpretation and reported phenotypic spectra [48]. Addressing this gap is essential for improving clinical interpretation and genetic counseling in this population. The present study aims to characterize CNVs affecting the 16p region in a cohort of patients with NDDs, and to delineate the spectrum of phenotypic variability as well as the potential genotype–phenotype correlations.

## 2. Materials and Methods

### 2.1. Patients

**Figure 1:**
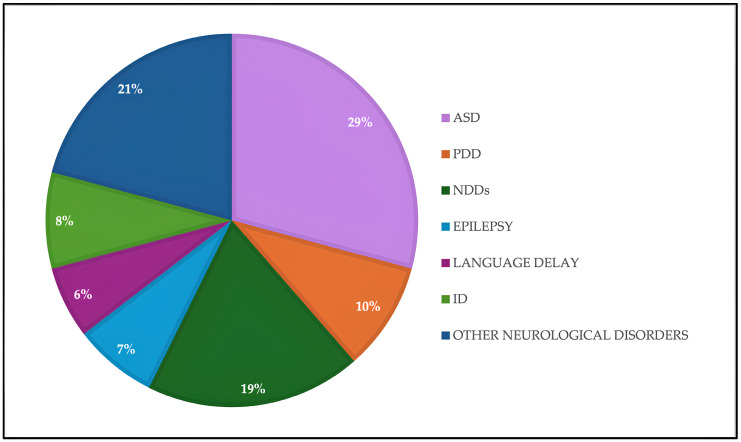
Distribution of diagnostic suspicions of the 1200 patients involved in the cohort study analyzed by a-CGH.

Between November 2023 and April 2025, we considered a cohort of 1200 pediatric and adolescent patients (all with postnatal diagnosis) with an average male-to-female ratio of 4:1, affected by neurodevelopmental disorders, who underwent genetic analysis at the Molecular Diagnostics Laboratory of CEINGE Biotecnologie Avanzate Franco Salvatore, Naples. Patients exhibited significant phenotypic heterogeneity, and the distribution of diagnostic suspicions was broad and predominantly attributable to the neurobehavioral spectrum. This retrospective study included patients referred for genetic testing based on a primary diagnostic suspicion reported by the referring clinician: ASD in 350 patients (29%), NDDs in 225 patients (19%), language delay in 75 (6%), psychomotor developmental delay (PDD) in 113 patients (10%), intellectual disability (ID) in 100 patients (8%), epilepsy in 87 patients (7%), and a heterogeneous group of other neurological disorders not attributable to the main categories, such as psychosis, anxiety, dysmorphic features, hypotonia, isolated attention deficit-hyperactivity disorder (ADHD), and learning difficulties in 250 patients (21%) (Figure 1). Given the retrospective nature of the study, a comprehensive and standardized clinical re-evaluation of all patients was not possible. Patients were therefore classified according to the primary diagnostic suspicion indicated at the time of referral, which constituted the main inclusion criterion. Individuals lacking sufficient clinical information or referred for non-neurodevelopmental indications were not included in the analysis. It is important to emphasize that comorbidities are common in neurodevelopmental disorders and that individual patients may present with multiple overlapping phenotypic features; consequently, classification based on a single diagnostic category represents a simplification and may limit genotype–phenotype correlations. When available, parental testing was performed to assess CNV segregation. The study was conducted according to the guidelines of the Declaration of Helsinki and approved by the Ethics Committee of the Faculty of Medicine (authorization no. 193/06, 25 October 2006; amendment no. 193/06/ESES1, 1 October 2014). Written informed consent was obtained from all subjects or their legal guardians prior to genetic testing. Consent included authorization for the use of biological material for diagnostic purposes and, in anonymized form, for research and statistical analyses.

### 2.2. Array CGH Analysis

Patients’ DNA and their parents’ DNA, when available, were isolated from peripheral blood samples using the CSC Blood DNA Kit (Promega, Madison, WI, USA), according to the manufacturer’s instructions. DNA purity and concentration were determined with a NanoDrop 2000 spectrophotometer (ThermoFisher Scientific Inc., Bartlesville, OK, USA). Following extraction, the patients’ DNA and their parents’ DNA were processed using the Agilent protocol (Version 8.0, December 2019). Array comparative genomic hybridization (a-CGH) was performed using SurePrint G3 Human CGH Array 4 × 180 K (Agilent Technologies, Santa Clara, CA, USA), according to the manufacturer’s recommendation. The Agilent SurePrint G3 Human CGH Array 4 × 180 K includes 180,000 CGH 60-mer probes with an average spacing of 13 kb, allowing an average resolution of 25 kb. A-CGH experiments were performed using sex-matched reference DNA. The reference DNA is high-quality genotyped DNA from normal males and females, included in the Agilent Genome Labeling Kit. The array images were acquired by the Agilent laser scanner G2600D. Image files were quantified, and data were visualized by using Agilent’s Cytogenomics software version 5.4.0.11 (Agilent Technologies, Santa Clara, CA, USA). The ADM2 algorithm for CNVs was set in Agilent’s Cytogenomics software with a threshold of 6. Chromosomal copy number changes were defined by a minimum of three adjacent probes and considered duplicated or deleted when results exceeded the minimum absolute mean log2 ratio of 0.25, a parameter that depends on an estimated DLRS (Derivative Log Ratio Spread) value, a quality control parameter that differs across all samples. Nucleotide positions were referred to the Human Reference Sequence (GRCh38) Assembly Dec. 2013 hg38 of UCSC Genome Browser (http://genome.ucsc.edu/, 12 November 2025). Molecular karyotypes were described in accordance with ISCN 2016 (International System for Human Cytogenomic Nomenclature 2016, Karger; https://doi.org/10.1159/isbn.978-3-318-06861-0). The identified CNVs were classified according to the American College of Medical Genetics and Genomics (ACMG) standards and guidelines [24] and by the comparison with the following international databases: Database of Genomic Variants (DGV; http://dgv.tcag.ca/dgv/app/home, 12 November 2025), Database of Chromosomal Imbalance and Phenotype in Humans (DECIPHER; https://decipher.sanger.ac.uk/, 12 November 2025), ClinGen (http://dbsearch.clinicalgenome.org/search/, 12 November 2025), The Human Gene Database, GeneCards (https://www.genecards.org/, 12 November 2025), ClinVar (National Center for Biotechnology Information, U.S. National Library of Medicine; http://www.ncbi.nlm.nih.gov/clinvar/, 12 November 2025), and Online Mendelian Inheritance in Man (OMIM; https://www.omim.org/, 12 November 2025). A CNV is classified as benign when it is observed in more than 1% of the population and described in at least 3 subjects reported in the DGV database. A CNV is classified as uncertain when it contains genes whose function is unknown, it is observed in both healthy and affected individuals, and there is insufficient evidence for a more certain classification. A CNV was classified to be likely pathogenetic when variants are reported in a few cases with a similar phenotype or never reported but in genes causative of the clinical phenotype, or as a pathogenetic one when it is a variant well documented in the literature as causative of disease. The analysis was also carried out by evaluating the correspondence of the alterations with the main diagnostic suspicion indicated by the clinician, to confirm a genotype–phenotype correlation.

## 3. Results

### 3.1. Distribution of CNVs in the 16p Region

**Figure 2:**
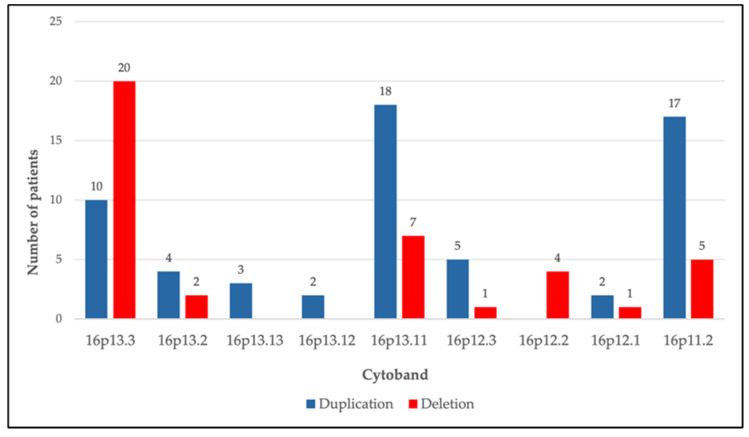
Graphical representation of the distribution of duplications and deletions across the 16p chromosomal region.

Our clinical study analyzed a cohort of 1200 patients with neurodevelopmental disorders who underwent genetic analysis by a-CGH, which allowed the identification of numerous CNVs. The focus was exclusively on variants located on the short arm of chromosome 16 (16p), and the data were analyzed and interpreted considering the available clinical, cognitive, and behavioral profiles. CNVs affecting the 16p region were identified in 96 patients (8% of the total cohort), including both deletions and duplications. We detected a total of 99 CNVs, classified according to the ACMG criteria: 5 CNVs with pathogenic clinical significance, 12 CNVs with likely pathogenic clinical significance, and 82 variants of uncertain clinical significance (VOUS). Overall, 60 duplications (60.6%) and 39 deletions (39.4%) were identified, distributed across the entire short arm of chromosome 16 (Figure 2). Grouping the alterations according to the region involved, the most frequent CNVs involve the chromosomal regions 16p13.3 (N = 30), 16p13.11 (N = 25), and 16p11.2 (N = 22), followed by less represented regions, such as 16p13.2 (n = 6), 16p12.3 (n = 6), 16p12.2 (n = 4), 16p13.13 (n = 3), 16p13.12 (n = 2), and 16p12.1 (n = 1) (Table 1).

### 3.2. Recurrent Alterations in the 16p Region

**Figure 3:**
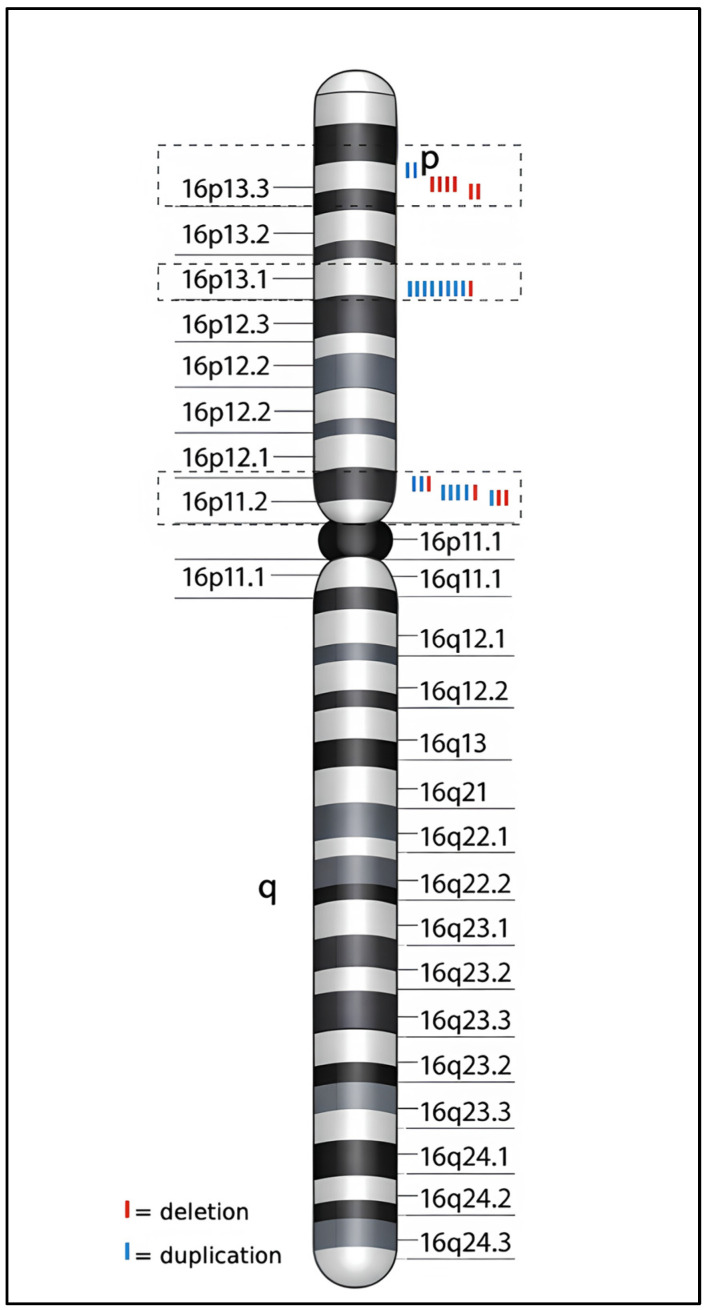
Distribution of recurrent CNVs identified in 16p13.3, 16p13.11, and 16p11.2 regions. The dotted outlines indicate regions of interest containing overlapping CNVs shared by different unrelated patients, represented by the blue/red bars.

CNVs identified in the 16p13.11 and 16p11.2 regions were mostly classified as pathogenic or likely pathogenic, in contrast to the other regions, where variants were mostly VOUS. Among the pathogenic and likely pathogenic alterations observed in our cohort, microdeletions and microduplications associated with known syndromes were identified, including proximal 16p11.2 microdeletion (16p11.2 proximal deletion syndrome, OMIM #611913), distal 16p11.2 microdeletion (16p11.2 distal deletion syndrome, OMIM #613444), 16p13.11 microdeletion (16p13.11 microdeletion syndrome, ORPHA:261236), and 16p13.11 microduplication (16p13.11 microduplication syndrome, ORPHA: 261243). Recurrent CNVs, i.e., alterations shared by multiple unrelated patients and characterized by overlapping nucleotide lengths, were also identified for each region, specifically, in the regions 16p13.3, 16p13.11, and 16p11.2 (Figure 3).

Three recurrent alterations in the 16p13.3 region have been identified. A 112.8-kb deletion at 16p13.3 (from nucleotide 6769601 to nucleotide 6882363) was identified in 4 male patients, while a 146.7-kb deletion at 16p13.3 (from nucleotide 6839407 to nucleotide 6986067) was found in 2 male patients. Both CNVs partially involve the *RBFOX1* gene (OMIM #605104). Lastly, a 71.4-kb duplication at 16p13.3 (from nucleotide 2610738 to nucleotide 2682131) was found to be overlapping in 2 patients (1 male and 1 female). In the 16p13.11 region, a “mini-cluster” consisting of 8 out of 25 patients emerged, all carrying the same 16p13.11 alteration (from nucleotide 14874998 to nucleotide 16198378), observed as a duplication in 7 cases (6 males and 1 female) and as a deletion in 1 case (female patient). The shared region has an extension of 1.3 Mb and involves OMIM genes, including the following: *MARF1* (OMIM #614593), *NOMO1* (OMIM #609157), *NDE1* (OMIM #609449), *ABCC6* (OMIM #603234), *ABCC1* (OMIM #158343), *MYH11* (OMIM #160745), *FOPNL* (OMIM #617149), *NTAN1* (OMIM #615367), *NPIPA1* (OMIM #606406), and *PDXDC1* (OMIM #614244). Three recurrent variants were also identified in the 16p11.2 region: a CNV of 545.6 kb from nucleotide 29641678 to nucleotide 30187279, present in 5 patients (4 duplications and 1 deletion); a CNV of 545.6 kb from nucleotide 29652999 to nucleotide 30198600, identified in 3 patients (1 duplication and 2 deletions). Furthermore, these CNVs are partially overlapping with each other and therefore both involve the following OMIM genes: *PRRT2* (OMIM #614386), *DOC2A* (OMIM #604567), *ALDOA* (OMIM #103850), *SEZ6L2* (OMIM #616667), *TAOK2* (OMIM #613199), *KCTD13* (OMIM #608947), *MAPK3* (OMIM #601795), and *CORO1A* (OMIM #605000). At last, a CNV of 217.3 kb from nucleotide 28813473 to nucleotide 29030797, also found in 3 patients (2 duplications and 1 deletion), involving *ATXN2L* (OMIM #607931), *SPNS1* (OMIM #612583), *SH2B1* (OMIM #608937), *ATP2A1* (OMIM #108730), *TUFM* (OMIM #602389), and *LAT* (OMIM #602354) OMIM genes. In our cohort, CNVs affecting chromosome 16p mapped to breakpoint intervals directly defined by the genomic alterations observed in the analyzed patients. Recurrent CNVs at 16p11.2 and 16p13.11 clustered within well-characterized breakpoint regions flanked by low-copy repeats (LCRs), consistent with a NAHR mechanism inferred from shared CNV boundaries. Specifically, the CNVs identified in our patients at 16p11.2 were in the proximal BP4–BP5 and distal BP2–BP3 intervals, while CNVs affecting 16p13.11 mapped to the BP1–BP2 region. Both deletions and duplications showed highly overlapping genomic coordinates among unrelated individuals, indicating recurrent breakpoint usage within the cohort. In contrast, CNVs detected at 16p13.3 exhibited variable sizes and non-canonical breakpoints, reflecting greater structural heterogeneity. Breakpoint architecture and CNVs distribution across chromosome 16p are summarized in Table 2.

The genomic analysis of each CNV was conducted with particular attention to genes involved in neurobiological processes or already associated with neurodevelopmental disorders. In addition to the genes previously mentioned, the following genes involved in other CNVs in the 16p region were also of particular interest: *DOC2A* (OMIM #604567), *KCTD13* (OMIM #608947), *QPRT* (OMIM #606248), *HIRIP3* (OMIM #603365), and *MVP* (OMIM #605088). The size of the CNVs is extremely heterogeneous, ranging from a few tens of kb to variants larger than 1 Mb, consistent with the predisposition of the 16p region to low-copy repeat (LCR)-mediated rearrangements.

**Figure 4:**
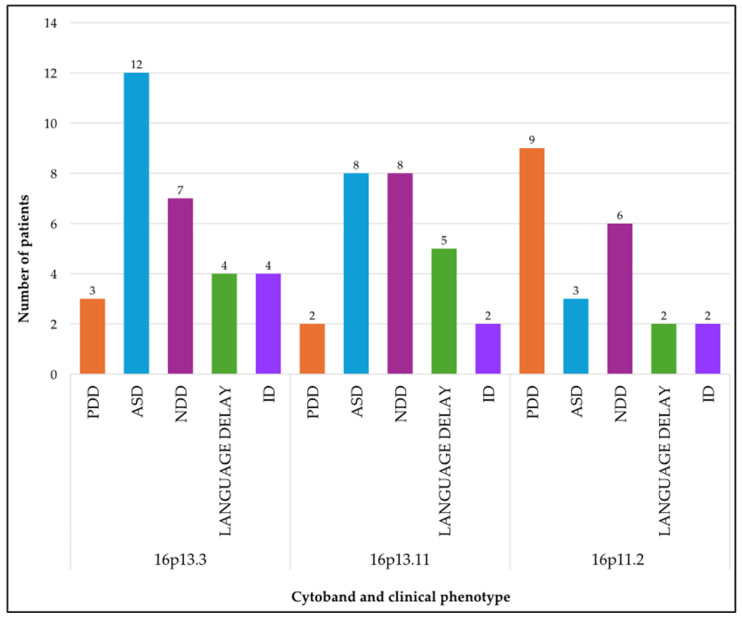
Phenotypic distribution of CNV carriers in the recurrent cytobands on chromosome 16: 16p13.3, 16p13.11, and 16p11.2 regions.

Based on our results, it was possible to perform a phenotypic stratification for patients with CNVs in the cytobands most prone to structural rearrangements. The association between identified CNVs and the phenotype of the patient in whom they were identified highlights that alterations in the 16p13.3 and 16p13.11 regions correlate primarily with an ASD and NDD phenotype, while CNVs in the 16p11.2 region are more frequently associated with PDD and NDD (Figure 4). Overall, the data highlight extensive genomic and phenotypic heterogeneity in the analyzed cohort, with the presence of recurrent clusters both at the cytogenomic (16p13.11, 16p11.2) and clinical levels (ASD, NDD, PDD). These findings provide the basis for an in-depth discussion of the role of CNVs on the short arm of chromosome 16 in neurodevelopmental disorders.

## 4. Discussion

In the present cohort of 96 patients with NDDs, analyzed by a-CGH, the 16p region emerges as a genomic locus of interest, previously reported to show high penetrance in neurodevelopmental disorders. Its intrinsically unstable architecture, characterized by widespread low-copy repeats (LCRs), predisposes it to recurrent structural rearrangements, likely through nonallelic homologous recombination (NAHR), as described in prior studies [41,49]. CNVs distributed along the 16p arm, encompassing both deletions and duplications of variable size, confirm the central role of this locus in the pathogenesis of complex neurodevelopmental phenotypes, including ASD, PDD, ID, and behavioral abnormalities, supporting the relevance of this locus without implying absolute causality [43,44,45]. Among these, 16p11.2 has been most frequently reported as clinically penetrant, with CNVs clustering within two well-characterized intervals: the proximal BP4–BP5 (~550–600 kb) and distal BP2–BP3 (~220 kb) regions [25,30,31]. In our cohort, BP4–BP5 rearrangements were mostly associated with ASD, language impairment, mild-to-moderate ID, and complex behavioral patterns, consistent with the previous studies [12,23]. In the context of this clinical-genetic profile, two representative cases illustrate the phenotypic impact of distal and proximal 16p11.2 deletions. The first case is an 8-year-old boy diagnosed with ASD according to the Diagnostic and Statistical Manual of Mental Disorders (DSM-5) (American Psychiatry, APA, Philadelphia, PA, USA, 2013) criteria [50]. Significant difficulties in social interaction, verbal and nonverbal communication, and restricted interests and repetitive behaviors had emerged since early childhood. We identified a pathogenic 217-kb distal microdeletion at the 16p11.2 (BP2–BP3) region. This rearrangement, consistently associated with ASD, developmental delay, and language impairment, includes the critical gene *SH2B1* and the adjacent genes *ATP2A1*, *TUFM,* and *LAT*. Among these, *SH2B1* emerges as the primary driver of the neurobehavioral phenotype, given its established role in hypothalamic and dopaminergic pathways and its strong association with ASD and executive dysfunction. A recent study on a Dutch cohort has further delineated the phenotype of BP2–BP3 deletion carriers, highlighting a high prevalence of ASD, marked motor delay, and behavioral and sleep problems [45]. The patient’s clinical presentation is fully consistent with the previously reported BP2–BP3 deletion phenotypes. The second case involves a 10-year-old boy with language impairment, executive dysfunction, and attentional difficulties, carrying a 546-kb proximal 16p11.2 microdeletion (BP4–BP5), classified as likely pathogenic. This interval encompasses a cluster of dosage-sensitive genes *PRRT2*, *MAPK3*, *TAOK2*, *DOC2A*, *KCTD13*, and *SEZ6L2*, involved in neuronal migration, synaptic development, and plasticity. The combined haploinsufficiency of these genes underlies the well-established 16p11.2 proximal microdeletion syndrome and supports its classification as a high-penetrance CNV for neurodevelopmental disorders. Conversely, distal BP2–BP3 deletions were associated with ASD, global developmental delay, language impairment, and behavioral dysregulation, in line with recent cohort studies [22,31,32,43,45,51,52]. These findings further support the distal 16p11.2 region as a critical neurodevelopmental hotspot with variable expressivity. A notable finding in our cohort is the marked male predominance among carriers of 16p11.2 deletions presenting with verbal apraxia [53]. This observation suggests the existence of a differential biological susceptibility in males to the neurobehavioral and neurolinguistic effects of chromosome 16 CNVs, arising in sex-specific vulnerability mechanisms, potentially involving hormonal modulation, neurodevelopmental timing, or differential compensatory capacity [54,55,56]. Notably, several dosage-sensitive genes within the 16p loci converge on neurodevelopmental pathways influenced by sex-specific factors. Genes such as *MAPK3* and *TAOK2* are involved in signaling cascades regulating neuronal migration and synaptic development, processes known to be modulated by sex hormones and sexually dimorphic neurodevelopmental trajectories. Similarly, *SH2B1* (16p11.2 distal) participates in hypothalamic and dopaminergic signaling, pathways showing sex-dependent organization, while *NDE1* (16p13.11) regulates neuronal progenitor proliferation and cortical development, biological processes displaying sex-specific vulnerability. Together, these data support a model in which sex acts as a biological modifier of the neurodevelopmental impact of 16p CNVs, contributing to the male-biased clinical expressivity observed in our cohort [43,57,58].

The 16p13.11 locus also has significant biological and clinical relevance with a distinct clinical profile, characterized by incomplete penetrance and broad phenotypic variability. According to several studies [38,59], most carriers exhibited mild or subclinical manifestations, although language impairment, epilepsy, and cardiovascular anomalies were also observed in our cohort. So, CNVs affecting the 16p13.11 region represent a recurrent neurodevelopmental susceptibility locus. Both microdeletions and microduplications, typically spanning 0.8–1.5 Mb and arising through NAHR-mediated mechanisms mediated by the presence of LCRs in the region [60], converge on dosage alterations of the *NDE1* gene, the principal candidate gene of the interval [61,62,63]. The *NDE1* gene plays a critical role in neuronal progenitor proliferation, cortical migration, and laminar organization, and its dysregulation is associated with developmental delay, language impairment, executive and attention deficits, epilepsy, and, less frequently, autism spectrum features. Clinical observations from our cohort are consistent with the previous evidence, as exemplified by a 13-year-old female patient carrying a 1.3-Mb microdeletion, classified as likely pathogenetic in the 16p13.11 region. She was diagnosed with epilepsy, difficulty in concentration, mild impairment of executive functions, and occasional episodes of emotional instability. The deleted region includes several genes with high brain expression, including *NDE1*. Several clinical cohorts have documented a significant increase in the frequency of this microdeletion in epileptic patients compared to the general population, suggesting that vulnerability to seizures derives precisely from the microcircuitry disorganization associated with reduced *NDE1* dosage [38,60,64]. Similarly, *NDE1* haploinsufficiency contributes to the cognitive and behavioral variability described in subjects with 16p13.11 microdeletion, including attention difficulties, executive function disorders, language delay, and, less frequently, autism spectrum traits [38,64]. In addition to *NDE1*, other genes included in the microdeletion, such as *NTAN1*, involved in neuronal protein turnover, and *MYH11*, a potential modifier gene, may contribute to the phenotype through synergistic mechanisms, consistent with a multi-hit genomic model frequently proposed for 16p13.11 CNVs in the literature [5,38,61,64]. The 16p13.11 region is also affected, although less frequently, by microduplications encompassing multiple genes involved in brain development and cardiovascular regulation, most notably *NDE1*, miR-484, and *MYH11* genes. Cohort studies report wide inter- and intra-familial variability, with psychomotor delay and mild-to-moderate intellectual disability representing the most common features, often accompanied by language impairment, learning difficulties, behavioral abnormalities, and autistic traits [38,39,42,65,66]. Consistent with these observations, we describe an 18-month-old girl presenting with global developmental delay, hypotonia, absence of expressive language, and impaired fine motor skills, in whom a-CGH identified a likely pathogenic 800-kb microduplication at the 16p13.11 region. The duplicated interval includes *NDE1*, supporting its role as the principal dosage-sensitive gene, as suggested in the literature [67,68]. Importantly, both reduced and increased *NDE1* dosage resulted in neurodevelopmental phenotypes, underscoring its strict dosage sensitivity and supporting a model in which balanced expression is required for normal cortical development. This observation is in line with the prior studies and does not imply definitive causality in our cohort.

Although less extensively studied, the 16p13.3 region also shows potential clinical relevance involving the *RBFOX1* gene associated with psychomotor delay and language impairment, consistent with the prior studies. Given its association with ASD and related neurodevelopmental phenotypes, *RBFOX1* could further expand the spectrum of dosage-sensitive genes contributing to 16p-associated disorders [69]. Additional CNVs involving less recurrent regions of chromosome 16p (16p12.2, 16p12.3, 16p13.12, 16p13.13) were associated with heterogeneous yet clinically relevant phenotypes, including hypotonia and neuromotor impairment. Given the small number of cases, genotype–phenotype correlations for these CNVs remain tentative, and these genes likely act as modulators, especially when present alongside more recurrent 16p variants. In our cohort, the coexistence of rare CNVs with recurrent rearrangements such as 16p11.2 or 16p13.11 was associated with increased phenotypic severity, in agreement with the two-hit model proposed by Girirajan et al. [29]. This framework supports the marked phenotypic variability observed among individuals with identical 16p CNVs and reinforces the view of chromosome 16p as a central neurogenetic hub. Here, recurrent high-penetrance CNVs, rare modulatory variants, and secondary genomic hits interact through dosage-dependent and combinatorial mechanisms, giving rise to a continuous spectrum of neurodevelopmental phenotypes shaped by broader genetic and epigenetic contexts. Overall, our findings reinforce the view of chromosome 16p as a central neurogenetic hub, where recurrent, highly penetrant CNVs (16p11.2, 16p13.11, 16p13.3), non-recurrent modulating variants, and extra-16p second hits contribute to defining a complex phenotypic continuum, supported by dose-dependent mechanisms, combinatorial effects, and epigenetic modulations [29,41,49,70]. Neurodevelopmental disorders associated with 16p rearrangements therefore arise not from linear gene–phenotype relationships, but from the integrated effects of genomic architecture, gene dosage sensitivity, and broader genetic background.

## 5. Conclusions

The results of this study on a cohort of Italian patients support the pathogenetic relevance of CNVs affecting the short arm of chromosome 16 (16p), a genomic hotspot of structural instability. The CNVs identified in this region show characteristics typical of loci most susceptible to rearrangements, including recurrence, variable penetrance, association with complex neuropsychiatric phenotypes, and involvement of numerous genes with critical functions in neurocognitive development. The integration of genomic data obtained by a-CGH and the phenotypic observations of patients confirmed the importance of considering the 16p region as a central functional node in the pathogenesis of NDDs. This evidence underscores the need to interpret each CNV not as an isolated event, but within a broader individual genomic context, which includes interactions among genes, regulatory mechanisms, modulating variants, and possible multi-locus events. However, this study is limited by its retrospective design, modest cohort size, and reliance on clinical reference data. Indeed, the phenotypic characterization was based on the primary diagnostic suspicion rather than standardized assessments, which may influence genotype–phenotype correlations. The moderate recurrence of some CNVs and incomplete parental testing further limit conclusions regarding penetrance and causality. Therefore, although our observations are consistent with the previously reported associations, they should be interpreted as descriptive and hypothesis-generating rather than definitive evidence of causality.

The conclusions are limited to the descriptive findings of this cohort. While high-resolution genomic technologies and sequencing methods have the potential to further elucidate NDD mechanisms, such applications are beyond the scope of the present study.

In summary, our findings provide a refined characterization of 16p CNVs in an Italian cohort and highlight the value of integrating genomic and clinical data for understanding genotype–phenotype correlations. Future research, leveraging advanced molecular platforms and larger cohorts, may expand these observations and support personalized diagnostic approaches.

## Figures and Tables

**Table 1:** Summary table of the distribution of alterations identified in patients in the different cytobands of the 16p chromosomal region involved and differentiated based on the type of CNV (deletion/duplication).

CNV on Chr16 ^1^	Number of Patients	Duplication	Deletion
16p13.3	30	10	20
16p13.11	25	18	7
16p11.2	22	17	5
16p13.2	6	4	2
16p12.3	6	5	1
16p12.2	4	0	4
16p13.13	3	3	0
16p13.12	2	2	0
16p12.1	1	1	0

^1^ Chr, chromosome.

**Table 2:** Summary table of breakpoint architecture and CNVs distribution across chromosome 16p region.

Cytoband	Genomic Coordinates (hg38) *	CNV Type (n. Patients)	Size	Canonical Breakpoints	OMIM Genes Involved	Associated Conditions
16p13.3	6769601_6882363	Del (4 p.)	112.8 kb	Non-canonical	*RBFOX1*	RBFOX1-related neurodevelopmental disorders
	6839407_6986067	Del (2 p.)	146.7 kb	Non-canonical	*RBFOX1*	RBFOX1-related neurodevelopmental disorders
	2610738_2682131	Dup (2 p.)	71.4 kb	Non-canonical	Not reported	Not reported
16p13.11	14874998_16198378	Dup (7 p.) Del (1 p.)	1.3 Mb	BP1–BP2	*MARF1*, *NOMO1*, *NDE1*, *ABCC6*, *ABCC1*, *MYH11*, *FOPNL*, *NTAN1*, *NPIPA5*, *NPIPA1*, *PDXDC1*	NDE1—lissencephaly and microcephaly (OMIM #614019), microhydranencephaly (OMIM #605013); ABCC6—pseudoxanthoma elasticum (OMIM #264800); MYH11—familial thoracic aortic aneurysm (OMIM #132900)
16p11.2	29641678_30187279	Dup (4 p.) Del (1 p.)	545.6 kb	BP4–BP5	*PRRT2*, *DOC2A*, *ALDOA*, *SEZ6L2*, *TAOK2*, *KCTD13*, *MAPK3*, *CORO1A*	PRRT2—paroxysmal disorders (OMIM #602066); 16p11.2 CNV syndrome (OMIM #611913)
	29652999_30198600	Dup (1 p.) Del (2 p.)	545.6 kb	BP4–BP5	*PRRT2*, *DOC2A*, *ALDOA*, *SEZ6L2*, *TAOK2*, *KCTD13*, *MAPK3*, *CORO1A*	PRRT2—paroxysmal disorders (OMIM #602066); 16p11.2 CNV syndrome (OMIM #611913)
	28813473_29030797	Dup (2 p.) Del (1 p.)	217.3 kb	BP2–BP3	*ATXN2L*, *SPNS1*, *SH2B1*, *ATP2A1*, *TUFM*, *LAT*	SH2B1—obesity, behavior features; ATP2A1—Brody myopathy (OMIM #601003)

* Coordinates are reported on the GRCh38/hg38 assembly. Recurrent CNVs are defined as overlapping genomic alterations identified in ≥2 unrelated patients. Gene lists are derived from the described shared intervals; p, patient; Del, deletion; Dup, Duplication; and BPs, Breakpoints.

## Data Availability

Data are contained within the text. Further details can be required to corresponding author.

## Abbreviations

The following abbreviations are used in this manuscript:

NDDs	Neurodevelopmental disorders
CNVs	Copy number variants
ASD	Autism spectrum disorder
ID	Intellectual disability
PDD	Psychomotor developmental delay
ADHD	Attention deficit-hyperactivity disorder
a-CGH	Array comparative genomic hybridization
NAHR	nonallelic homologous recombination
LCRs	Low-copy repeats
BP	Breakpoint
